# Spiro-strain Takes Chemiluminescence to Flash-Mode

**DOI:** 10.1021/acscentsci.4c00017

**Published:** 2024-02-15

**Authors:** Sujit Kumar Das, Ankona Datta

**Affiliations:** Department of Chemical Sciences, Tata Institute of Fundamental Research, 1 Homi Bhabha Road, Mumbai, 400005, India

We fondly remember fun times with glowsticks whenever we think of chemiluminescence. The chemistry behind glowsticks, in which a chemical reaction between two molecules releases energy to excite a dye that relaxes via long, sustained visible emission,^1^ is often used in school science outreach to familiarize children with luminescence. In a recent issue of *ACS Central Science*, Shabat, Houk and co-workers take the slow-glow of chemiluminescence to flash-mode.^2^ How did the authors achieve this and why is it important to take chemiluminescence to flash-mode?

**Figure 1:**
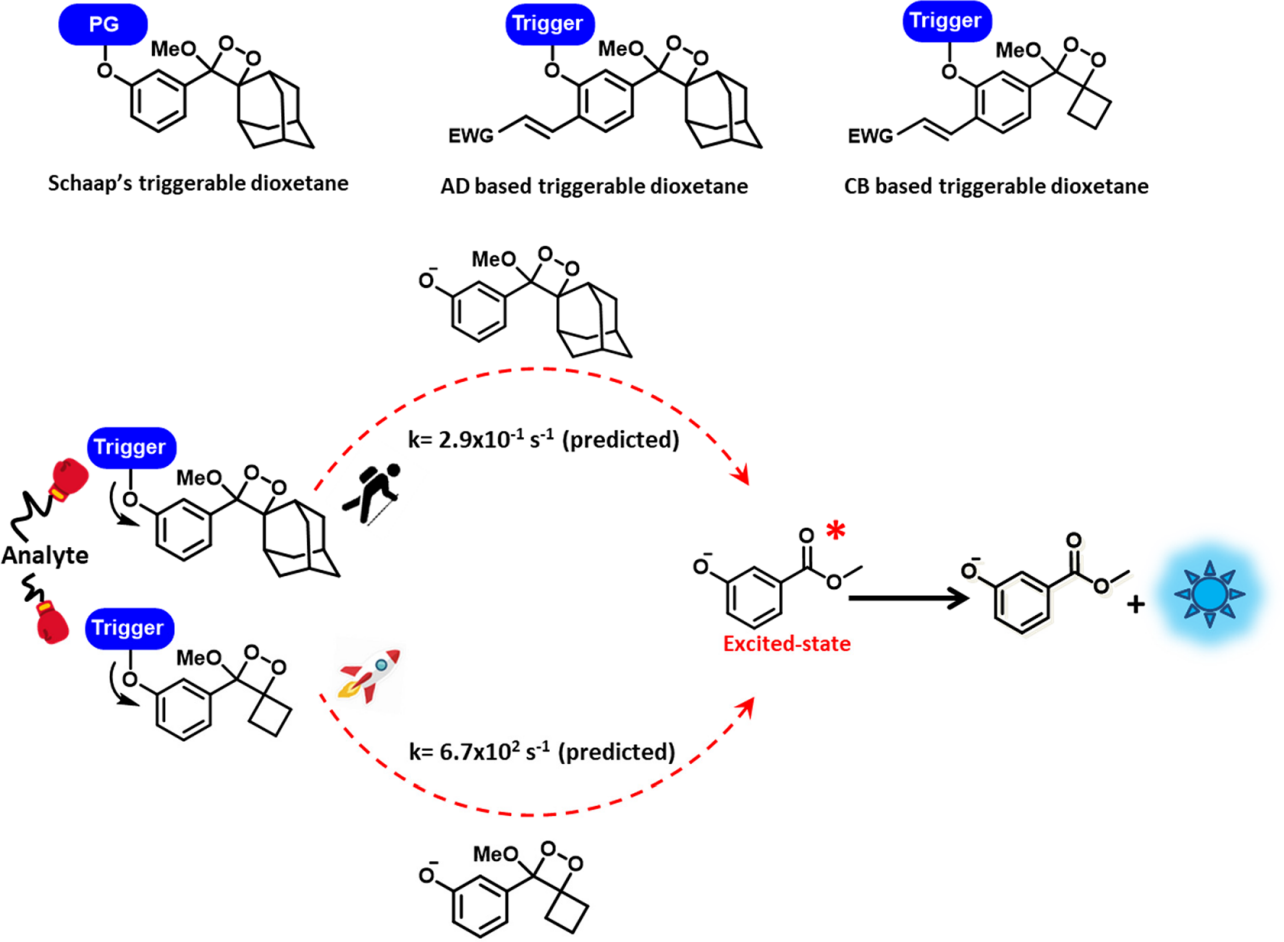
Top panel, chemical representation of Schaap dioxetanes (left), adamantane (AD) based dioxetanes (middle), and cyclobutyl-based (CB) strained dioxetanes (right). Bottom panel depicts the rate enhancement obtained due to spiro-strain in CB-based strained dioxetanes.

The invention of single-component chemiluminescent molecules was an important development in the luminescent probe field and forms the foundation of this work. Schaap and co-workers developed chemiluminescent molecular probes containing a protecting group (Figure 1).^3^ Removal of the protecting group led to a rapid electron transfer reaction releasing a “chemi-excited” molecule. This invention opened the gateway to replacing the protecting group with a trigger that could be activated only through a specific stimulus like the presence of a chemical entity, bioanalyte, or enzyme.^4^ Thus, chemiluminescence entered the sensing arena.

**Figure 2:**
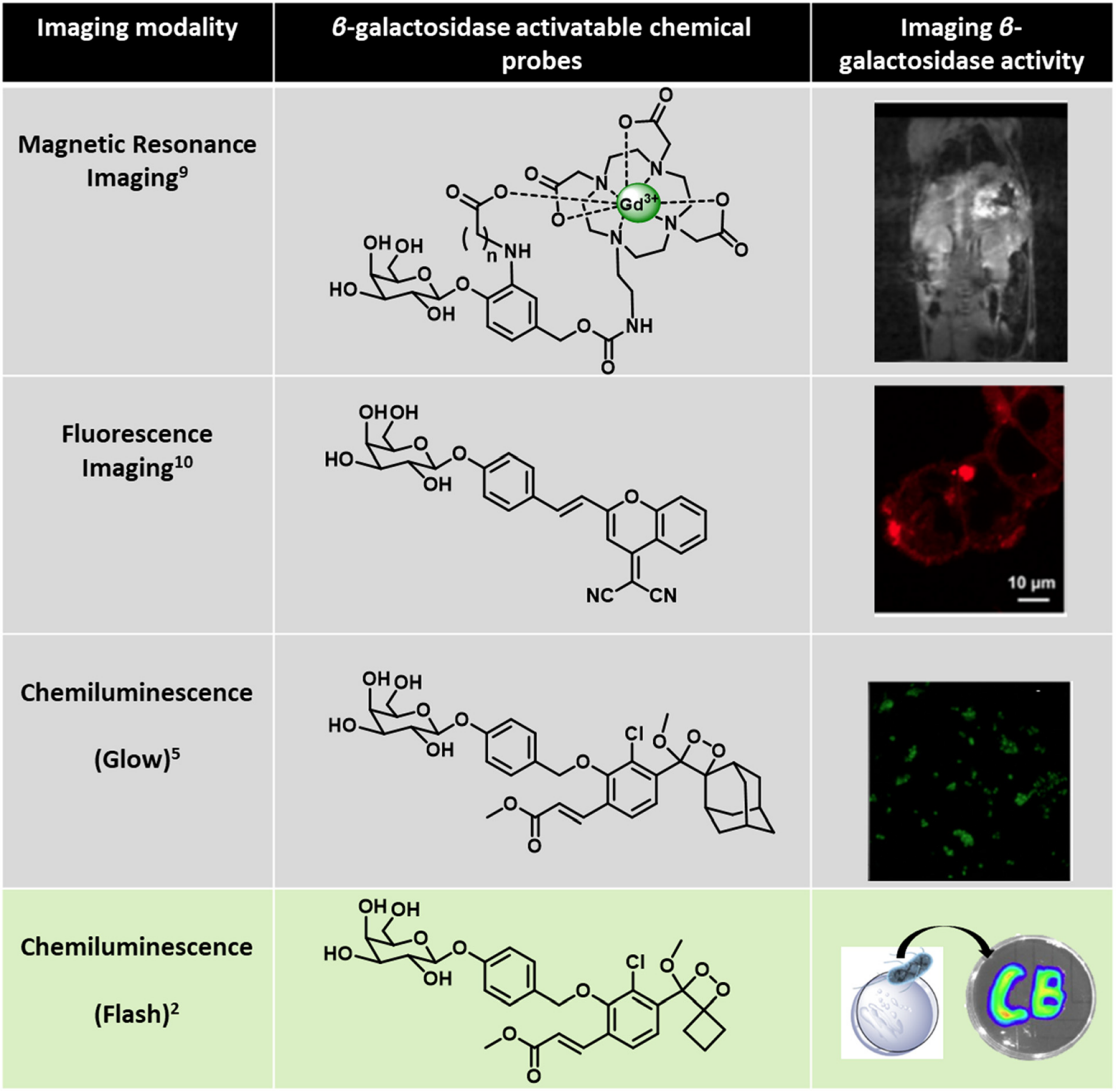
β-Galactosidase triggered probes in different imaging modalities and their applications in various biological contexts. A magnetic resonance “smart probe” images β-galactosidase activity in a mouse model (top panel), reproduced with permission from ref (9). Copyright 2019 John Wiley and Sons. Fluorescence-based self-immolative probe affords near-infrared turn on imaging of β-galactosidase activity in *lacZ*-(+) 293T cells with β-galactosidase overexpression (second panel from top), reproduced with permission from ref (10). Copyright 2016 American Chemical Society. Chemiluminescence microscopy image of *lacZ*-(+) HEK293T cells depicting β-galactosidase activity (third panel from top), reproduced with permission from ref (5). Copyright 2017 American Chemical Society. Flash probe for rapid detection of β-galactosidase activity in *E. coli* (bottom panel), reproduced with permission from ref (2). Copyright 2023 American Chemical Society.

However, molecules developed by Schaap, phenoxy-1,2-dioxetanes (Figure 1), while highly emissive in organic solvents, did not luminesce in water and, hence, biological applications were limited.^3^ In an important improvement, Shabat and co-workers modified the Schaap design by introducing an electron-withdrawing group leading to probes with long sustained emission in water (Figure 1).^5^ This allows chemiluminescence to work in aqueous biological environments leading to applications in sensing biomolecules within living systems (Figure 2).^5,6^

But one issue still remained. Although chemiluminescent probes could report on the presence of an analyte in a cellular system via imaging in a microscopy setup, due to the slow-sustained emission of the probes temporal information was completely lost. Further, an attractive feature of single-component chemiluminescent molecules was potential applications in ex-vivo detection of chemical pollutants and pathogens. However, low emission intensities over long time periods remained a key drawback deterring these applications.

In this work, Shabat and co-workers set forth to solve this temporal issue.^2^ Using insights from previous computational studies that had illuminated the mechanism of chemiluminescence in phenoxy-1,2-dioxetanes, they reckoned that the slowest step in the chemi-excitation reaction was the cleavage of the O–O bond in the dioxetane.^7,8^ Based on this insight, a series of strained cyclobutyl-dioxetanes were designed (Figure 1). They envisioned using a fused-cyclobutane-dioxetane unit. The O–O bond cleavage via electron transfer from the phenoxide to the dioxetane unit would release the strain in the “spiro-fused” moiety thereby increasing the rate of “chemi-excitation”. Computational studies performed by the authors confirmed the hypothesis, following which the authors convincingly showed that the rate of chemiluminescence could be increased by 100 times compared to the parent adamantane containing probe (Figure 1). Due to this enhanced rate, light-emission from the modified probe occurred in a flash, leading to significantly higher intensity over a shorter period of time (s–min).

This strategic development was creatively used to detect the activity of an enzyme β-galactosidase in *E. coli* cells. The β-galactosidase-triggered chemiluminescent probe allowed the instantaneous detection of bacteria with high sensitivity when a solution containing the probe was sprayed on an agar plate containing bacteria. As few as ∼5 × 10^5^ bacterial cells could be detected under an imaging setup. This experiment highlights the potential of the new spiro-fused chemiluminescent probes in rapid detection of environmental pollutants and pathogens. To put this experiment into perspective, galactosidase activity has been previously detected via fluorescence and magnetic resonance imaging modalities (Figure 2).^9,10^ Here, the authors use this previously optimized and popular assay to illustrate an application of their probe and importantly highlight the enhanced detection sensitivity.

Single-component chemiluminescent probes come with a unique advantage of minimal technical requirements for imaging applications, since these molecules do not need a laser source for excitation.^4^ Biological signaling processes occur in ms–s time scales, and we are only limited by our imagination as to the numerous exciting biological realms that will become accessible if the temporal responses of these probes are taken to the ms time scales.
